# Stigma as a barrier to suicide prevention efforts in Iran

**DOI:** 10.3389/fpubh.2022.1026451

**Published:** 2023-01-09

**Authors:** Maryam Masoomi, Seyedehmahboobeh Hosseinikolbadi, Fahimeh Saeed, Vandad Sharifi, Amir Hossein Jalali Nadoushan, Sheikh Shoib

**Affiliations:** ^1^Department of Psychiatry, Tehran University of Medical Sciences, Tehran, Iran; ^2^Suicide Prevention Committee, Iranian Psychiatric Association, Tehran, Iran; ^3^Psychosis Research Center, University of Social Welfare and Rehabilitation Sciences, Tehran, Iran; ^4^Department of Psychiatry, School of Medicine, Tehran University of Medical Sciences, Tehran, Iran; ^5^Mental Health Research Center, Department of Psychiatry, Psychosocial Health Research Institute, School of Medicine, Iran University of Medical Sciences, Tehran, Iran; ^6^Department of Psychiatry, Jawahar Lal Nehru Memorial Hospital, Srinagar, India; ^7^Sharda University, Greater Noida, Uttar Pradesh, India; ^8^Mind Wellness Center, Srinagar, India

**Keywords:** stigma, suicide prevention, suicide behavior, mental health, Iran

## Abstract

Suicide and suicide attempt affect a considerable part of the general population, and in spite of their prevalence, the stigma associated with suicide remains an unsolved problem surrounding this important public health problem, especially in lower-income countries such as Iran. Evidence shows that help-seeking from formal mental health services for suicidal people is low in countries like Iran. Previous studies on Iranian survivors of suicide attempts have shown that these people experience fear of stigma due to labels such as loss of faith in God, having forms of severe mental illnesses (“*madness*”), and being involved in unaccepted sexual relationships. The associated stigma prevents them from seeking appropriate health and social services. Although both self-stigma and public stigma contribute to an unwillingness to seek mental health care and suicide prevention efforts in Iran, public stigma may be of greater consequence, significantly impeding an individual's likelihood of accessing care for their suicidal thoughts or attempts. In such circumstances, many people with suicidal thoughts miss out on social and formal support programs offered by social and healthcare providers. In this perspective article, focusing on the public stigma regarding suicide in Iranian society, we address the challenges and barriers to seeking suicide prevention efforts in Iran and discuss culturally appropriate strategies to improve the current situation.

## 1. Introduction

Suicide is a critical public health issue caused by various individual, economic, and sociocultural factors. More than 700,000 suicide deaths occur worldwide each year, and most of these incidents happen in low- and middle-income countries (LMICs) (1). Although the suicide rate among Islamic societies is low, evidence shows that the trend of suicide deaths in Iran is increasing; In the past years, it has increased to 9.9 per 100,000 people (2). Due to cultural and socioeconomic factors, men in Iran are particularly vulnerable (3). In addition, family conflicts and marital issues are the leading reported triggers of suicide, followed by financial difficulties and poor academic performance (1).

Suicide is one of the mental health issues that is accompanied by stigma, and numerous studies have documented highly prevalent stigmatizing attitudes toward suicidal individuals especially in low- and middle-income countries like Iran (4, 5).

Individual Attitudes, beliefs, and behavioral patterns can be affected by the stigma of mental illness and suicide. Important aspects of stigma, such as fear and shame, negative beliefs toward mental illness, prejudice from society, social isolation, and discrimination, serve as obstacles to people who are at risk of suicide and prevent them from seeking assistance (6). It should be noted that seeking appropriate help is critical to preventing the exacerbation of mental health problems and reducing the risk of suicide yet, according to one study, only 62% of people who attempt suicide sought mental health services in the year leading up to it (7). Reducing the stigma surrounding mental health problems is linked to the decline of several negative mental health outcomes, notably suicide (8). The first step in reducing public health problems such as suicide is improving public awareness about suicide (9). Legal actions, such as the establishment of government anti-discrimination laws to reduce stigma around suicide and boost help-seeking, are also crucial in lowering suicide attempts. These include increasing the public and policy-makers' understanding of suicide (10, 11). We explore the difficulties and barriers to suicide prevention efforts in Iran in this perspective paper, which focuses on the public stigma associated with suicide in Iranian society. We also review culturally appropriate improvement strategies.

## 2. Suicide stigma and challenges in Iran

A key barrier to using mental health services in Iran is the stigma associated with psychiatric problems (12). The interactive phases of problem assessment, assistance-seeking decision, and service selection are often how people seek out healthcare treatments for mental health. However, several factors, such as mental health literacy, attitudes and beliefs regarding suicide, the perceived need for treatment, and other internal and external impediments, May reduce the likelihood of an individual engaging in these help-seeking behaviors (13, 14).

In Iran, utilizing mental health services is hampered by the widespread stigmatization of mental illnesses and, in general, psychiatry practice among the general public, and even among educated people and authorities. Therefore, stigma-related shame and the fear of being labeled psychiatric diagnosis are significant obstacles to obtaining care (12, 15). According to some Iranian research, inadequate mental health literacy exists not only in the general population but also among students and the educated. Some scholars may refer to psychiatric disorders as spiritual problems that should be dealt with spiritually (16, 17). This is despite the fact that there is an inverse relationship between mental health literacy and stigma, in such a way that the lower the mental health literacy in a society, the greater the stigma in that society (16). This not only becomes a barrier to help-seeking among vulnerable people in society, but also affects the quality of life in the community. In addition, poor mental health literacy in Iranian society has led to self-stigma among different populations and is an important barrier to help-seeking in high-risk individuals (18).

People who have experienced indifference and/or negative attitudes from healthcare workers, may use health services inadequately (15). In Iranian medical education system, despite the obligatory psychiatric rotations, the mental health education, stigmatization and its consequences do not parallel in attention toward physical health (19). Interestingly, increasing literacy about suicide among nurses leads to improved attitudes toward people who attempt suicide (18). This may ameliorate people's willingness to use mental health services.

Another significant component that may heighten or lessen social stigma is culture (19). In terms of stigma, the three main facets of culture are media, literature, and art. They are crucial in the stigmatization (or destigmatization) of mental health problems, suicidal thoughts and attempts, and those who have lost loved ones to suicide (9, 20). Iranians have a sense of humor and frequently crack jokes in response to current events and pressing social issues. Jokes that have a negative outlook on people with mental illnesses can enhance stigma (21). In addition, expressions like “if...(Something happens), I shall kill myself” are frequently used in talks and jokes. In affluent nations, one of the main objectives of culturally-based suicide prevention efforts has been to reduce stigma by raising public awareness about suicide and the adverse consequences of stigmatizing attitudes (22).

Suicide attempts and behaviors are strongly prohibited in Islam; Suicide is an unpardonable sin that guarantees a person's immediate entrance into damnation and hell. Suicide is consequently highly stigmatized in nations with a majority of Muslim population, such as Iran. Other religious societies are not exempt (23).

There are no adequate comprehensive and effective efforts in Iran to increase knowledge and awareness about mental disorders, people with mental illnesses and suicide. Sometimes the media also provoke very negative attitudes toward mental illnesses and suicide. World Health Organization has disseminated the Media Guideline for Reporting Suicide, however, Iranian online media like many other LMICs do not fully follow such recommendations (24). In addition, authorities in many countries are reluctant to report the true rate of suicide attempts and deaths, and this can be an important barrier to comprehending this complex problem, resulting in a misunderstanding of people's needs and reduced help-seeking for preventive services in high-risk populations (25). Moreover, underreporting of suicide attempts and deaths can result from the community's denial or reluctance to attribute them to suicide. That underreporting of this information can have an impact on the provision and funding of essential suicide prevention services that may facilitate a reduction in suicide behavior and death.

Previous qualitative research indicates that personal unfavorable attitudes and views of Iranian policymakers and health authorities may have influenced their decisions to not fully implement a comprehensive stigma reduction strategy (12, 25). Another barrier to improving mental health literacy and creating and implementing prevention interventions in this area is a lack of financial resources. The financial resources are vital to sustain mental health programs or upgrade pilot programs to national level. The cost of services, lack of insurance coverage for suicide and its complications, are financial difficulties that people who have attempted suicide face to directly (26).

## 3. Suicide prevention efforts and challenges in Iran

In LMICs such as Iran, suicide is considered a public health problem that can be managed not only through mental health approaches, but also through comprehensive community-based and social programs. Implementation of community-based screening programs in countries such as Japan and the United States has been reported to be effective and safe in improving treatment referrals and utilization of suicide prevention services (27, 28). Accordingly, about three decades ago, the mental health integration program in Iran's primary health care (PHC) system was evaluated and implemented (29). Furthermore, Iran's national suicide prevention program was integrated into the PHC (30). In line with such initiative, a large number of clinical psychologists have been recruited to health centers affiliated with the PHC system in recent years, and play an effective role in reducing suicide (31). In the PHC, almost every client is screened for suicidal ideation by community health workers or General Practitioners (29). However, in Iran, we may need a comprehensive screening and referral program not only in primary care settings, but in public and educational settings, especially for at-risk individuals such as adolescents and young adults. However, it should be noted that, concurrent with developing suicide screening programs around the country, health professionals should be adequately educated to provide the best response to people who are at risk of suicide.

In recent years, Reform in the mental health care system has led to Iran's Comprehensive Mental and Social Health Services (the SERAJ Program) which has been implemented in some areas of the country (32, 33). Community Mental Health Centers (CMHCs) are an integral part of the program and provide services for the local community. Among CMHC's services is collaborative care with primary care providers in the health centers; the aim is to improve detection and treatment of mental disorders as well as suicide. Also, CMHCs provide aftercare services for people with severe mental disorders after discharge from the hospital, and rehabilitation services by psychoeducation and skill training (32–36).

Although suicide hotlines are an essential component of every system for preventing suicide (37), there is no national suicide prevention hotline in Iran. The State Welfare Organization of Iran offers free telephone consultations and a few medical universities provide hotline services, for all mental health concerns (38). However, to the best of our knowledge, there is no published report on number of suicidal individuals who use these free services and their effectiveness on suicide prevention.

The mental health system faces some challenges in the field of suicide prevention. One of the most obvious ones is the insufficient-allocated budget, both in health care and research. Insufficient budget leads to poor quality of care and poor access to services across the population. Consequently, effective pilot programs may not be implemented at the national level. For implementation at the national level, it needs to be integrated into the national health care services/programs. This has proved to be very difficult, if not impossible, for several reasons; among which are negative attitudes of health care policymakers toward mental disorders, insufficient governmental health care budgets, bureaucratic hurdles of integrative efforts, unsustainable resources, features of innovations that make them hard to scale, inadequate training and support staff, etc. (39). For example, in a small community trial in three districts in Iran, SERAJ was associated with improved mental health literacy and decreased prevalence of mental disorders in the intervention districts (33). After 6 years, the program has been expanded to 23 districts, however, the SERHJ program has not yet been fully scaled to a national level, despite the promising outcomes during the trial (34).

Though, this is not limited to Iran or other developing countries; it is observed in high-income countries as well; for example, Bégin et al. (40) call Canada a “country of perpetual pilot projects”.

In Iran, mental health prevention programs, especially community-based ones, are not adequate for the needs of the community. More importantly, the effective strategies foreseen in these programs, such as improving the quality and quantity of the services provided, enhancing the quality of health record taking, reducing the social stigma related to public awareness, managing media reporting, and limiting access to suicide methods, have not yet been implemented at scale. Often, the cultural aspect of these programs has been neglected and there has not been proper awareness at the community level to increase mental health literacy. In addition, weak intersectoral cooperation among organizations leads to a significant reduction in the effectiveness of the programs (26, 29, 32).

Even though mental health research suffers from inadequate funding in recent years, some evidence-based suicide prevention interventions have been carried out in Iran (41–45). However, many of these studies suffer from important methodological shortcomings that limit their applicability at the community level, such as being quasi-experimental without follow-up, being conducted on military forces personnel, or having limited sample sizes. A comprehensive community-based suicide prevention and intervention program that is supported by a range of relevant organizations (such as the Ministry of Health, Welfare Organization, and Ministry of Education, Ministry of Science) at the national level is urgently needed.

## 4. Discussion

Suicide is a significant and complex public health problem that needs to be carefully addressed in all its facets, including efforts to raise awareness among the society, health professionals, and policymakers, the development of a comprehensive, multi-level, and multi-directional national program, the provision of adequate funding and insurance support for mental health care, and the training of skilled professionals. Multi-level suicide prevention efforts that integrate different approaches may yield the best outcomes, but such a strategy in a country like Iran is still in its infancy, and there is much to learn and research about different suicide prevention programs and how to effectively combine them to achieve a successful model. The main challenge for research and practice involves identifying the most efficient ways to reach vulnerable people who may not benefit from current prevention and awareness programs (46). However, there are many obstacles to identifying optimal solution. Universality of stigma is the main obstacle that remains unsolved in Iran, and across the globe. Although both self-stigma and public stigma contribute to unwillingness to seek suicide prevention efforts in Iran (21), public stigma (such as gender-related myths in society, prohibition of holding funerals for suicide victims, negative judgment of people who have died by suicide as weak persons unable to cope with their problems, etc.) may have more negative effects on people and cause them to hide their suicidal thoughts and attempts. In such circumstances, many people with suicidal thoughts miss out on social and formal support programs offered by health activists and healthcare providers (21).

More recent Iranian studies have shown that most of the general population has become familiar with mental illnesses through the media and especially movies (15, 19). However, mass media in Iran, as a cultural representation of the community, does not provide information about mental health, and this can create negative attitudes toward people with mental illness. In countries such as Germany and the United States, media campaigns seek to reduce the stigma of mental health problems, increase suicide awareness, and increase help-seeking behaviors (9). Focusing on the stigma of mental health problems, these campaigns seek to change public beliefs about people with mental disorders. They also aim to improve attitudes toward mental health services and treatment. Help-seeking behaviors and attitudes play an important role in the use of services for mental illnesses. A related meta-analysis reported progressive negative attitudes toward help-seeking over the past decades, possibly due to the medicalization of mental health problems (47). In Iran, multiple centers make decisions that can affect the mental health of the public and on many occasions they do not collaborate well. Although the Ministry of Health and Medical Education is considered the leading organization responsible for public mental health, it has limited budgets and power to take care of mental health of the whole country. Therefore, in Iran, we need the cooperation of organizations and authorities to develop suicide prevention campaigns and encourage non-governmental associations to implement the formulated strategies.

Religious and cultural factors should be considered to reduce suicide stigma, which calls on experts in these fields to work together. There is an initiative in Indonesia in which religious leaders attempted to de-stigmatize mental disorders and suicide in order to establish a national suicide prevention strategy (48). Similar measures can be taken in Iran as a Muslim country. In addition, we should not forget important informal sources of support (i.e., family and friends) to de-stigmatize suicidal thoughts and normalize help-seeking. By providing proper education, these sources can help develop the protective actions in an individual's life by assisting the person to reinforce supportive relations and life skills. These sources provide support and listen to people experiencing suicide, understand their feelings, talk to them, encourage them to seek and adhere to preventive treatments and help them stay safe in crises (49, 50).

We have provided some recommendations to reduce suicide stigma and encourage utilization of suicide prevention programs in the society and government levels in Table 1. Since suicide can be prevented, we urgently need to change the attitude of the public and policymakers through national educational programs. We also need to adapt appropriate strategies for suicide prevention focusing on protective factors, such as cultural strengths, family ties, spirituality, etc.

**Table 1:** Recommendations to reduce the stigma toward suicide and encouraging utilizing suicide prevention system.

**Public level recommendations**
1- Addressing stigma of psychiatric disorders and suicide by concentrating on vulnerable groups such those with mental illnesses, the youth, women, elders, and those who are struggling financially. 2- Trying to increase knowledge about mental health. Discussing one's own mental pain and thoughts of suicide but avoiding condemning those who have attempted suicide 3- Boosting public awareness of mental health through social media, instructions at schools and universities, and public gathering places like religious services or festivals. 4- Supporting the rights of those who suffer from mental disorders by social activism. Also, Social Influencers' revelation of their own suicidal experiences and how they overcame them can be helpful.
**Policymakers**
1- Creating strong networks between government agencies and authorities, and non-profit organizations that work to prevent suicide to save money, eliminate duplication of efforts, increase cooperation between organizations, improve coordination of care, and increase implementation capacity. 2- Maintaining policies to ensure the confidentiality and privacy of people receiving mental health services, these policies should primarily target insurance companies. 3- Promoting mental health services will lead to effective suicide prevention. It might be accomplished by raising the level of expertise of PHC mental health professionals, requiring insurance companies to pay health consequences of suicide, and offering affordable, efficient treatment approaches that are based on the most recent research for mental disorders. 4- Creating a national suicide referral system and hotline. 5- Requiring media outlets to adhere to and monitor WHO reporting guidelines for suicide. The use of hotlines and mobile applications can be promoted in suicide prevention advertising efforts on social media and platforms like Instagram, offering dependable and useful support for those at risk of suicide. 6- Suicide prevention methods should be modified to account for local culture and ecosystem. It implies that the suicide prevention system needs to be revised for susceptible groups, such as mental health professionals or communities with distinctive characteristics. 7- Providing consumers with educational material could be a crucial intervention strategy. These readily available alternatives would be helpful to a lot of people, but in order to best serve the wide user base, these programs would need to be modified to include different languages, cultural perspectives, and geographic features (51). 8- Iranian Early Career psychiatrist might encourage a national and international cooperative network to create studies and provide guidelines to reduce stigma associated with mental illnesses and to launch new strategies for promoting mental health. 9- To examine the suicide as a public health problem and to develop powerful preventative measures, a mix of qualitative and quantitative research is suggested.

## Data availability statement

The raw data supporting the conclusions of this article will be made available by the authors, without undue reservation.

## Author contributions

MM and FS: conceptualization and writing first draft. SH, VS, AJ, and SS: critical revising and writing of the final version. All authors contributed to the article and approved the submitted version.

## References

[1] Mirhashemi S Motamedi MHK Mirhashemi AH Taghipour H Danial Z. Suicide in Iran. Lancet. (2016) 387:29. 10.1016/S0140-6736(15)01296-9 26766345

[2] Hassanian-Moghaddam H Zamani N. Suicide in Iran: the facts and the figures from nationwide reports. Iran J Psychiatry. (2017) 12:73. 28496505 PMC5425355

[3] Izadi N Mirtorabi SD Najafi F Nazparvar B Nazari Kangavari H Hashemi Nazari SS. Trend of years of life lost due to suicide in Iran (2006–2015). Int J Public Health. (2018) 63:993–1000. 10.1007/s00038-018-1151-1 30074055

[4] Al-Shannaq Y Aldalaykeh M. Suicide literacy, suicide stigma, and psychological help seeking attitudes among Arab youth. Curr Psychol. (2021) 2021:1–13. 10.1007/s12144-021-02007-9 34177209 PMC8214717

[5] Shoib S Chandradasa M Saeed F. Armiya'u AYu Roza TH Ori D. Suicide, stigma and COVID-19: a call for action from low and middle income countries. Front Psychiatry. (2022) 13:894524. 10.3389/fpsyt.2022.894524 35845441 PMC9283681

[6] Carpiniello B Pinna F. The reciprocal relationship between suicidality and stigma. Front Psychiatry. (2017) 8:35. 10.3389/fpsyt.2017.00035 28337154 PMC5340774

[7] Noorani N Alavi K Malakooti SK Salimi S Jalali A. Mental health services use among people that attempt suicide by taking a drug overdose during the last year before their suicide commission. Int J Rev Life Sci. (2017) 7:80–5.

[8] Lally J. ó Conghaile A, Quigley S, Bainbridge E, McDonald C. Stigma of mental illness and help-seeking intention in university students. Psychiatrist. (2013) 37:253–60. 10.1192/pb.bp.112.041483

[9] Niederkrotenthaler T Reidenberg DJ Till B Gould MS. Increasing help-seeking and referrals for individuals at risk for suicide by decreasing stigma: the role of mass media. Am J Prev Med. (2014) 47:S235–S43. 10.1016/j.amepre.2014.06.010 25145745

[10] Clark W Welch SN Berry SH Collentine AM Collins R Lebron D. California's historic effort to reduce the stigma of mental illness: The Mental Health Services Act. Am J Public Health. (2013) 103:786–94. 10.2105/AJPH.2013.301225 23488486 PMC3698820

[11] Kim EJ Yu JH Kim EY. Pathways linking mental health literacy to professional help-seeking intentions in Korean college students. J Psychiatr Ment Health Nurs. (2020) 27:393–405. 10.1111/jpm.12593 31954091

[12] Taghva A Farsi Z Javanmard Y Atashi A Hajebi A Khademi M. Stigma barriers of mental health in Iran: A qualitative study by stakeholders of mental health. Iran J Psychiatry. (2017) 12:163. 29062367 PMC5640577

[13] Gould MS Greenberg T Munfakh JLH Kleinman M Lubell K. Teenagers' attitudes about seeking help from telephone crisis services (hotlines). Suicide Life Threat Behav. (2006) 36:601–13. 10.1521/suli.2006.36.6.601 17250466

[14] Tay S Alcock K Scior K. Mental health problems among clinical psychologists: stigma and its impact on disclosure and help-seeking. J Clin Psychol. (2018) 74:1545–55. 10.1002/jclp.22614 29573359

[15] Tavakoli S Sharifi V Taj M Mohammadi MR. Stigma of depression and its relationship with attitudes toward seeking professional help among students. Adv Cogn Sci. (2010) 12:19–33.31954091

[16] Jafari A Nejatian M Momeniyan V Barsalani FR Tehrani H. Mental health literacy and quality of life in Iran: a cross-sectional study. BMC Psychiatry. (2021) 21:1–11. 10.1186/s12888-021-03507-5 34641793 PMC8507341

[17] Mahmoodi SMH Ahmadzad-Asl M Eslami M Abdi M Kahnamoui YH Rasoulian M. Mental health literacy and mental health information-seeking behavior in Iranian University Students. Front. Psychiatry. (2022) 13:893534. 10.3389/fpsyt.2022.893534 35770063 PMC9234209

[18] Gholamrezaei A Rezapour-Nasrabad R Ghalenoei M Nasiri M. Correlation between suicide literacy and stigmatizing attitude of nurses toward patients with suicide attempts. Revista Latinoamericana de Hipertensión. (2019) 14:351–5.

[19] Sayarifard A Ghadirian L Mohit A Eftekhar M Badpa M Rajabi F. Assessing mental health literacy: What medical sciences students' know about depression. Med J Islam Repub Iran. (2015) 29:161. 26000256 PMC4431358

[20] Fu K-W Chan Y-Y Yip PS. Newspaper reporting of suicides in Hong Kong, Taiwan and Guangzhou: compliance with WHO media guidelines and epidemiological comparisons. J Epidemiol Commun Health. (2011) 65:928–33. 10.1136/jech.2009.105650 20889589

[21] Azizpour M Taghizadeh Z Mohammadi N Vedadhir A. Fear of stigma: The lived experiences of Iranian women after suicide attempt. Perspect Psychiatr Care. (2018) 54:293–9. 10.1111/ppc.12237 29165826

[22] Hegerl U Althaus D Schmidtke A Niklewski G. The alliance against depression: 2-year evaluation of a community-based intervention to reduce suicidality. Psychol Med. (2006) 36:1225–33. 10.1017/S003329170600780X 16707028

[23] Shoib S Armiya'u AYu Nahidi M Arif N Saeed F. Suicide in Muslim world and way forward. Health Sci Rep. (2022) 5:e665. 10.1002/hsr2.665 35702511 PMC9178353

[24] Arafat S Ahmad A Saeed AK Feizi O Saeed F Menon V. Quality of online media reporting of suicidal behavior in Iran during COVID-19 pandemic in Reference to the World Health Organization Guidelines. Global Psychiatry Arch. (2022) 5:70–6. 10.52095/gpa.2022.4883.1047

[25] Adib M Esmaeili M Zakerimoghadam M Nayeri ND. Barriers to help-seeking for elder abuse: A qualitative study of older adults. Geriatr Nurs. (2019) 40:565–71. 10.1016/j.gerinurse.2019.04.003 31076204

[26] Yoosefi Lebni J Abbas J Khorami F Khosravi B Jalali A Ziapour A. Challenges facing women survivors of self-immolation in the Kurdish regions of Iran: a qualitative study. Front Psychiatry. (2020) 11:778. 10.3389/fpsyt.2020.00778 32922314 PMC7456816

[27] Oyama H Koida J Sakashita T Kudo K. Community-based prevention for suicide in elderly by depression screening and follow-up. Commun Ment Health J. (2004) 40:249–63. 10.1023/B:COMH.0000026998.29212.17 15259630

[28] Gould MS Marrocco FA Hoagwood K Kleinman M Amakawa L Altschuler E. Service use by at-risk youths after school-based suicide screening. J Am Acad Child Adolesc Psychiatry. (2009) 48:1193–201. 10.1097/CHI.0b013e3181bef6d5 19858758 PMC2891889

[29] Bolhari J Ahmadkhaniha H Hajebi A Yazdi SAB Naserbakht M Karimi-Kisomi I. Evaluation of mental health program integration into the primary health care system of iran. Iran J Psychiatry Clin Psychol. (2012) 17.

[30] Rezaeian M Platt S Arensman E. Iran's national suicide prevention program: opportunities, challenges, and next steps. Crisis J Crisis Interven Suicide Prevent. (2022) 43, 344–7. 10.1027/0227-5910/a000788 34042463

[31] Zalsman G Hawton K Wasserman D van Heeringen K Arensman E Sarchiapone M. Suicide prevention strategies revisited: 10-year systematic review. Lancet Psychiatry. (2016) 3:646–59. 10.1016/S2215-0366(16)30030-X 27289303

[32] Damari B Sharifi V Asgardoon MH Hajebi A. Iran's comprehensive mental and social health services (SERAJ program): a pilot protocol. Iran J Psychiatry. (2021) 16:116. 10.18502/ijps.v16i1.5387 34054991 PMC8140297

[33] Hajebi A Sharifi V Asgardoon MH Damari B. The effectiveness of the pilot implementation of iran's comprehensive mental and social health services (the SERAJ Program): a controlled community trial. Iran J Psychiatry. (2021) 16:168. 10.18502/ijps.v16i2.5818 34221043 PMC8233554

[34] Sharifi V Hajebi A Damari B Mohammadjafari A. Specialized outpatient services: Community Mental Health Centers (CMHCs). Iran J Psychiatry. (2021) 16:87. 10.18502/ijps.v16i1.5383 34054987 PMC8140302

[35] Damari B Sharifi V Asgardoon MH Hajebi A. The community action program works to improve mental health at the district level: the evaluation of the community action program in districts of Iran. Iran J Psychiatry. (2021) 16:451. 10.18502/ijps.v16i4.7233 35082858 PMC8725181

[36] Damari B Sharifi V Asgardoon MH Hajebi A. Community Action Package in Iran's Comprehensive Mental and Social Health Services (the SERAJ Program). Iran J Psychiatry. (2021) 16:76. 10.18502/ijps.v16i1.5382 34054986 PMC8140293

[37] Organization WH. Preventing suicide: a resource for establishing a crisis line. World Health Organization. (2018).

[38] Counseling Psychological Affairs. The State Welfare Organization of Iran. (2021). Available from: https://en.behzisti.ir/news/35511/Counseling-and-psychological-affairs (accessed December 21, 2022).

[39] Wakida EK Talib ZM Akena D Okello ES Kinengyere A Mindra A. Barriers and facilitators to the integration of mental health services into primary health care: a systematic review. Syst Rev. (2018) 7:1–3. 10.1186/s13643-018-0882-7 30486900 PMC6264616

[40] Bégin HM Eggertson L Macdonald N. A country of perpetual pilot projects. CMAJ. (2009) 180:1185. 10.1503/cmaj.090808 19506272 PMC2691427

[41] Anisi J Rahmati Najarkolaei F Esmaeeli A Hagghi A. Evaluate the effect of problem solving skills to reduction of suicidal ideation of soldiers. Ebnesina. (2014) 16:42–6. 10.30505/7.4.147

[42] Mousavi SG Zohreh R Maracy MR Ebrahimi A Sharbafchi MR. The efficacy of telephonic follow up in prevention of suicidal reattempt in patients with suicide attempt history. Adv Biomed Res. (2014) 3:198. 10.4103/2277-9175.142043 25337528 PMC4202499

[43] Goudarzi AH Golmohammadi AA Bashirgonbadi S Samadi S. Effectiveness based on reducing stress (MBSR) on suicidal thoughts and aggression in the soldiers with normal task force of Malek-e-Ashtar Arak Garrison training course. J Police Med. (2018) 7:147–52.

[44] Zahedi AM Khedri B. Effectiveness Problem-Solving Skills Training to Reduce the Suicide Thought of Soldiers. (2015).

[45] Malakouti SK Nojomi M Ghanbari B Rasouli N Khaleghparast S Farahani IG. Aftercare and Suicide Reattempt Prevention in Tehran, Iran. Crisis. (2022) 43:18–27. 10.1027/0227-5910/a000754 33563037

[46] Vijayakumar L. Challenges and opportunities in suicide prevention in South-East Asia. WHO South-East Asia J Public Health. (2018) 6:30–3. 10.4103/2224-3151.206161 28597856

[47] Mackenzie CS Erickson J Deane FP Wright M. Changes in attitudes toward seeking mental health services: A 40-year cross-temporal meta-analysis. Clin Psychol Rev. (2014) 34:99–106. 10.1016/j.cpr.2013.12.001 24486521

[48] Onie S. Indonesian National Suicide Prevention Strategy 2022: A Preliminary Report. (2022).

[49] Edwards TM Patterson JE Griffith JL. Suicide prevention: The role of families and carers. Asia-Pacific Psychiatry. (2021) 13:e12453. 10.1111/appy.12453 33666375

[50] Frey LM Cerel J. Risk for suicide and the role of family: a narrative review. J Fam Issues. (2015) 36:716–36. 10.1177/0192513X13515885

[51] Ramnath S Suri G. Managing depression in India: Opportunities for a targeted smartphone app. Int J Soc Psychiatry. (2021) 67:1035–45. 10.1177/00207640211032253 34348491

